# Visual perspective taking and laterality decisions: Problems and possible solutions

**DOI:** 10.3389/fnhum.2013.00549

**Published:** 2013-09-06

**Authors:** Mark May, Mike Wendt

**Affiliations:** ^1^Spatial Cognition Research Unit, Helmut-Schmidt-University, Hamburg, Germany; ^2^Experimental Psychology Unit, Helmut-Schmidt-University, Hamburg, Germany

**Keywords:** spatial cognition, embodiment, visual perspective taking, mental transformation, own-body-transformation task, laterality tasks, spatial S-R compatibility, agency

## Abstract

Perspective taking plays an important role in different areas of psychological and neuroscientific research. Visual perspective taking is an especially prominent approach generally using one of two experimental tasks: in the own-body-transformation task observers are asked to judge the laterality of a salient feature of a human figure (e.g., is the glove on the left or right hand?) from the figure’s perspective. In the avatar-in-scene task they decide about the laterality of objects in a scene (e.g., is the flower on the left or right?) from the avatar’s point of view. Increases in latencies and/or errors are interpreted as originating from additional cognitive processes predominately described as observer-based perspective transformations. A closer look reveals that such an account is disputable on grounds related to the use of laterality judgments. Other transformation accounts, i.e., object or array transformations, as well as non-transformational accounts, i.e., extra processing due to spatial conflicts, have not been adequately considered, tested, or ruled out by existing research. Our review examines visual perspective tasks in detail, identifies problems and makes recommendations for future research.

## INTRODUCTION

Research on human perspective taking is gaining momentum as can be seen by the increasing number of experimental studies in different research areas, such as spatial reasoning, mental imagery, life-span cognitive development, theory of mind, empathy, aviation research, and teleoperations. The different fields have in common that they want to come up with accounts of the cognitive mechanisms underlying the ability to mentally switch into and spatially act from perspectives that are not our own, and sometimes those of others.

Two fundamentally different lines of research on spatial perspective taking can be distinguished: Research on mental perspective taking uses memory-based testing methods. In one line of work, participants first learn a layout of objects and are then asked to point to the previously learned objects without being able to look at the scene while bodily or imaginally switching into various perspectives. Measures of geometric differences between learned, body-defined and to-be-imagined perspectives have been found to be good predictors of pointing latencies and errors, and results are used to test competing processing accounts (e.g., Rieser, 1989; Easton and Sholl, 1995; Shelton and McNamara, 1997; Creem-Regehr, 2003; May, 2004; Avraamides and Kelly, 2008). Other studies examine mental perspective taking by using language, graphics, or maps as learning input and with other testing procedures (e.g., De Vega et al., 1996; Bryant and Tversky, 1999; Avraamides, 2003; Sohn and Carlson, 2003).

Research on visual perspective taking, on the other hand, uses perception-based testing methods. Participants usually look at a visual display including a human figure, and have to decide about the side of a critical feature of the figure while adopting the figure’s perspective (OBT or own-body transformation task; e.g., Parsons, 1987), or about relative object positions from the figure’s point of view (AIS or avatar-in-scene task; e.g., Amorim, 2003). Although both tasks are usually treated separately in the literature, the majority of OBT- and AIS-studies have in common that they use laterality decisions, i.e., observers have to make left or right judgments about absolute or relative object locations from the figure’s point of view. Recently, the number of behavioral and neurophysiological studies on visual perspective taking has been growing (Blanke et al., 2005; Creem-Regehr et al., 2007; Kessler and Thomson, 2010; Yu and Zacks, 2010; Dalecki et al., 2012 and others reviewed here). Note, that studies on viewpoint-dependent object (Tarr and Bülthoff, 1998) or scene recognition (Diwadkar and McNamara, 1997) are not considered, as their focus is on memory-based identification processes, and not on perspective taking.

The overall picture of findings on visual perspective taking is complex. In general, one finds increases in response times and errors the larger the spatial difference between the observer’s and the figure’s spatial perspective. This is taken to reflect additional cognitive processes described as observer-based perspective transformations (PT). In contrast to this widely held view, our review will argue that alternative accounts, e.g., object transformations (OT) of the figure in the OBT-task, or array transformations (AT) in the AIS-task, have been brought forth, but so far have not been systematically evaluated and pursued. Furthermore, the review will show that combining visual perspective taking tasks with laterality judgments leads to spatially compatible and incompatible responses, with consequences that have not been adequately addressed up to now.

## TASKS AND BASIC FINDINGS

### OWN-BODY TRANSFORMATION TASK

**FIGURE 1:**
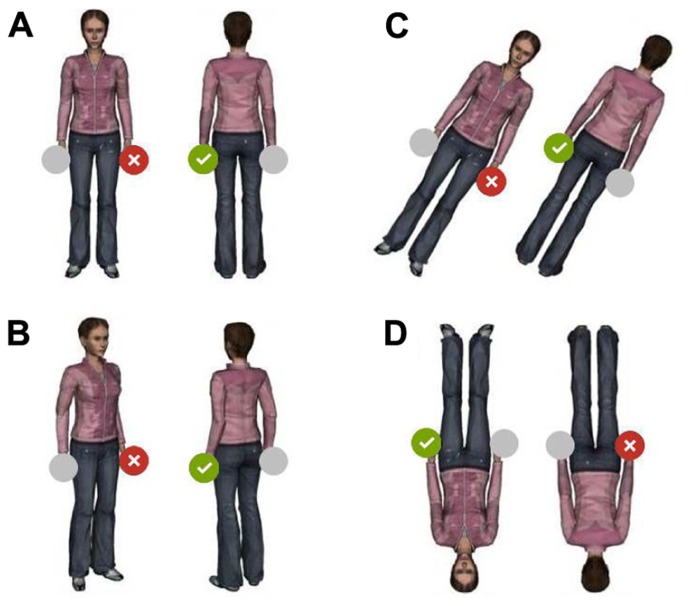
**Examples of OBT-stimuli.** The task of the observer is to decide whether the figure’s left or right hand is highlighted by a critical feature (green or red disc), and to press a corresponding left- or right-hand key. Various stimuli and features (e.g., human or abstract figures, gloved hand, ball or disc in hand) are used in actual experiments. Left side: Different upright figure stimuli (**A** and **B**) with compatible (tick mark) and incompatible (cross) correct responses. Right side: Different figures with rotations of 30° and 180° in the picture plane (**C** and **D**) with compatible (tick mark) and incompatible (cross) correct responses. Only figures with the critical feature on the figure’s left hand are shown; compatibilities are the same for features on the figure’s right hand.

Experiments using the OBT task show an isolated human figure with a salient body feature (e.g., a glove, a hand-held ball or disk). The observer’s task is to decide whether the salient feature is on the left or right as seen from the figure’s point of view and to respond by pressing a left- or right-hand key (or using another response indicating left and right). **Figure 1** shows examples of OBT-stimuli.

Consistent with the notion that observers mentally transform their own perspective until it matches the figure’s perspective before deciding about laterality, responses usually are faster for back-facing figures, i.e., when figures look in the same direction as the observer, compared to front-facing figures, i.e., when observer and figure look in opposite directions (Parsons, 1987; Zacks et al., 1999; Blanke et al., 2005; Jola and Mast, 2005; Arzy et al., 2006, 2007; Mohr et al., 2006, 2010; Gardner and Potts, 2010, 2011; Thakkar and Park, 2010; Braithwaite et al., 2011; Steggemann et al., 2011; Gardner et al., 2012; Gronholm et al., 2012; May and Wendt, 2012). No such performance differences are found when observers have to decide about the laterality of the critical feature from their own perspective (referred to as which-side-task), rather than from the avatar’s perspective (Blanke et al., 2005; Gardner and Potts, 2010, 2011; Braithwaite et al., 2011; Gardner et al., 2012). In support of a PT account of these findings, more than half of the participants report to switch into the avatar’s perspective when solving the task (Parsons, 1987; Zacks and Tversky, 2005; Gronholm et al., 2012).

### AVATAR-IN-SCENE TASK

**FIGURE 2:**
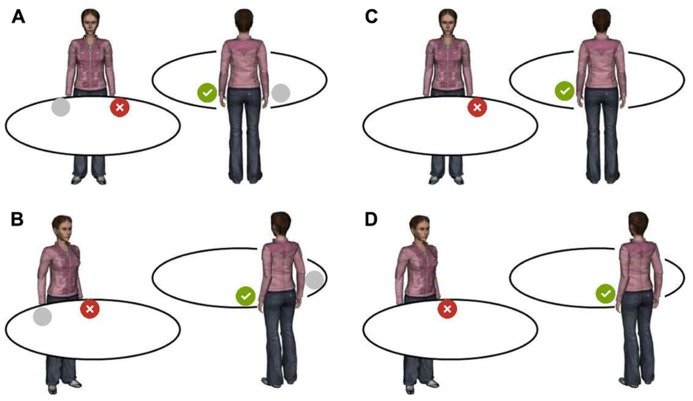
**Examples of AIS-stimuli.** The task of the observer is to decide whether the critical object (green or red disc) is on the right or left as seen from the avatar’s perspective, and to press a corresponding left- or right-hand key. Different scenes illustrate compatible (tick mark) and incompatible (cross) correct responses for different rotations of the avatar in the depth plane. Left side: Stimuli that request relative judgments **(A, B)**: Is the left or right stimulus from the avatar’s perspective the critical one? Right side: Stimuli that request absolute judgments **(C, D)**: is the critical object on the left or right side from the avatar’s perspective? Only figures with the critical object on the avatar’s left side are shown; compatibilities are the same for right-side objects.

Experiments using the AIS-task show an avatar (or a different symbol indicating the relevant perspective) looking at a spatial scene from varying angles of rotation in the horizontal plane. The observer’s task is to decide whether a critical object in the scene (e.g., flower) is on the left or right side from the avatar’s point of view. **Figure 2** provides examples of AIS-stimuli.

Response times for laterality judgments grow monotonically with the disparity of the avatar’s and the participant’s perspectives (e.g., Keehner et al., 2006; Michelon and Zacks, 2006; Kessler and Rutherford, 2010; Kessler and Thomson, 2010; Kockler et al., 2010). Similar to the back-facing advantage in the OBT task, these findings have been interpreted in terms of time to transform one’s own perspective into the avatar’s perspective.

## THEORETICAL ACCOUNTS OF VISUAL PERSPECTIVE TAKING

The above interpretations of OBT- and AIS-studies have been used to identify brain regions mediating visual perspective taking (e.g., Zacks et al., 1999, 2002; Blanke et al., 2005), and also to look into processing strategies used with human and non-human stimuli (e.g., Yu and Zacks, 2010). It turns out, however, that observed performances in laterality judgment tasks lead to difficulties when researchers try to interpret them as indicators of PTs. In the following, we look at existing evidence from the perspective of a PT-account, and at arguments used to defend it against competing OT/AT-accounts, or spatial compatibility explanations.

### CONFOUNDING SPATIAL TRANSFORMATIONS AND RESPONSE CONFLICTS

Under a variety of conditions, responses are faster and less error-prone when a target is presented at a location that spatially corresponds with the location of the requested response as compared to situations where the target location spatially corresponds with an incorrect response (Proctor and Vu, 2006). Dual-route models attribute such S-R compatibility effects to automatic activation of the spatially corresponding response along a processing route largely independent of intention-based S-R translation processes (Hommel, 1993; De Jong et al., 1994).

#### Spatial compatibility in OBT-tasks

**Figure 1** shows that the location of the target feature spatially corresponds with the correct response for the back-facing upright figure whereas it corresponds with the incorrect response, when the figure is shown front-facing. Spatial compatibility should facilitate responses to back-facing compared to front-facing figures. Intermediate orientations of the OBT-figure in the depth plane can be presumed to lead to graded compatibility effects.

#### Spatial compatibility in AIS-tasks

The AIS-task is in most aspects similar to the OBT-task, and similar problems arise (see **Figure 2**). On the one hand, and different from the centered presentation of OBT-stimuli, the positioning of the avatar-object-ensemble on the screen, can shift to the left or right from the screen’s center, potentially producing independent spatial (i.e., Simon-type) compatibility effects. On the other hand, and similar to the OBT-task, the relative position of the target object (left/right) within the ensemble as seen from the observer’s perspective, corresponds to the laterality of the correct response up to rotation angles of 90°. In contrast, rotations larger than 90° lead to a reversal of this situation, yielding spatial S-R-correspondence in the former case, and spatial non-correspondence in the latter case. Again, spatial compatibility and incompatibility should be maximal for avatars in the 0° (back-facing) and 180° (front-facing) positions, and might produce gradual effects for intermediate rotations. Both problems are rarely addressed in the literature.

#### Empirical evidence for spatial compatibility effects

Several findings are consistent with the assumption that OBT-performances are influenced by spatial compatibility. For instance, Gardner and Potts (2011, Exp. 1; Parsons, 1987, Exp. 2a) used vocal “left” and “right” responses, which are known to produce smaller compatibility effects than manual responses, and found a reduced back-facing advantage. Moreover, some manipulations which reversed the assigned correspondence values yielded a back-facing disadvantage. For instance, Arzy et al. (2006) asked participants to treat the depicted figure as a mirror reflection of their own body, and obtained slower responses for back-facing as compared to front-facing figures, while at the same time observing the well-known back-facing advantage with standard OBT task instructions.

Other studies presented the figure in different orientations in the picture plane, including upside-down versions, for which front-facing figures come with spatially corresponding, and back-facing figures with spatially conflicting responses (**Figure 1**). Upside-down presentation of figures either reduced (Steggemann et al., 2011), or even changed the back-facing advantage into a disadvantage (Parsons, 1987; Zacks et al., 1999; Jola and Mast, 2005; May and Wendt, 2012). Furthermore, Gardner and Potts (2011, Exp. 2) obtained a back-facing disadvantage by instructing participants to cross their hands and decide about laterality by key-presses with their corresponding hand, thereby reversing laterality and response locations (see, however, May and Wendt, 2012, for a back-facing advantage with uncrossed arms when left and right keys were labeled “right” and “left,” respectively).

Although this review focuses on perceptual laterality judgment tasks for which the confound of facing direction and compatibility is most obvious it should be noted that Simon-like spatial interference effects have also been found with respect to a remembered previous location of a current stimulus (e.g., Zhang and Johnson, 2004). More generally, the problem of spatial compatibility is also present in memory-based perspective taking tasks and has been subject of thorough discussion (e.g., May, 2004). Furthermore, perception- and memory-based tasks not asking for laterality decisions (e.g., color judgments) may induce spatial compatibility effects if the location of the response varies with respect to the same spatial dimension as the target stimulus feature (e.g., indicating red and green with left- and right-side key presses, respectively). Other tasks with non-spatial decision criteria (e.g., same-different, visibility, or numerosity judgments) could also induce spatial conflicts. In such cases it must be ensured that compatibility levels are balanced across facing directions.

#### Controlling for spatial compatibility

Attempts to control for compatibility have used figures with an outstretched arm across the body midline, where observers make laterality decisions regarding the outstretched arm. Although a back-facing advantage was also found for such figures (Parsons, 1987, Exp. 2b), this evidence is not conclusive, as it is possible that participants respond on the basis of a non-switching body feature such as the shoulder (Jola and Mast, 2005). Avoiding this problem, May and Wendt (2012) controlled for spatial compatibility by using horizontal figures (i.e., 90°-rotated) with hands equidistant to the figure’s upper and lower end; in spite of this arguably neutral conditions, a clear back-facing advantage was found (see also Parsons, 1987; Jola and Mast, 2005; Steggemann et al., 2011).

### LITTLE INDUBITABLE EVIDENCE FOR PERSPECTIVE TRANSFORMATIONS

Since the spatial end-state of PTs can principally also be reached by spatially equivalent OTs of the figure in the OBT-task, or ATs of the avatar-object-ensemble in the AIS-task, both constitute potential alternative explanations for the typical facing direction effects found in both tasks. OTs have been extensively studied in mental rotation research, by presenting a stimulus that differs in orientation from a second version of the same stimulus, or that is moved away from its canonical orientation, while asking participants to make a same/mirror-reversed judgment. Such studies show monotonic increasing reaction time (RT)-slopes for increasing rotation angles in both the picture and the depth plane (Shepard and Cooper, 1982).

#### Slope differences as evidence for PT

Slope differences play an important role in studies using OBT-tasks which try to distinguish between PT- and OT-accounts. These studies include rotations in the picture plane, and find slope differences for back- and front-facing figures. While **RTs increase with rotation angle for back-facing figures, slopes are strongly reduced, absent, or even reversed for front-facing figures (Parsons, 1987; Jola and Mast, 2005; Zacks and Tversky, 2005; Yu and Zacks, 2010; Steggemann et al., 2011; May and Wendt, 2012, Exp. 2; Zacks et al., 2000, 2002). Thus, performances for back-facing (but not for front-facing) figures are consistent with findings from research on mental rotation with same vs. mirror-reversed objects. The missing slopes in laterality decisions for front-facing figures have been repeatedly taken as evidence for PT-accounts (e.g., Yu and Zacks, 2010). For this argument to work, minimal costs for transformations in the picture plane have to be postulated. This constraint can be met by assuming that PTs are realized as shortest path spatial transformations; i.e., all rotation trajectories of observer-based switches into front-facing figures have the same rotation angle (i.e., 180°), irrespective of the figure’s orientation in the picture plane (see Parsons, 1987, p. 190).

#### Alternative explanations for slope differences

The observed slope differences for rotations in the picture plane can also be accounted for by compatibility assumptions (May and Wendt, 2012). Specifically, figures presented upside-down reverse S-R-compatibility values; i.e., compatible responses become incompatible, and vice versa. Applied to upside-down figures this means, that back-facing figures produce spatial conflicts, while front-facing figures do not (see **Figure 1**). Intermediate rotations of the upright figure in the picture plane, should lead to graded effects of compatibility.

#### PT- vs. OT-instructions

Independent support for PT-assumptions comes from experiments that use particular transformation instructions. Specifically, Zacks and Tversky (2005) observed positive RT-slopes for front-facing figures in a laterality judgment task when participants were asked to use object rotation strategies on the figures. However, near-zero slopes were found when participants received explicit PT-instructions or unspecific task instructions. Furthermore, averaged across all orientations of the figures in the picture plane substantial RT-increases for object-based instructions as compared to both observer-based transformation or unspecific instructions were found.

Although the findings of Zacks and Tversky (2005) can be interpreted to reveal that PTs are naturally used for human figures (if not instructed otherwise), in our opinion this does not provide indisputable evidence for PTs, as the following considerations show: explicit object rotation instructions (e.g., “imagine the figure rotating until it is upright,” p. 281) may induce OTs (i.e., picture-plane rotations of the front-facing figure) that are not the same OTs that can be assumed to be at work with unconstrained task instructions (i.e., shortest path object rotations of front-facing to back-facing figures). In other words, finding positive RT-slopes with explicit instructions to rotate the object in the picture plane speaks against the use of such OTs with non-specific instructions (i.e., flat slopes), but not against other types of OTs as a strategy spontaneously adapted by observers. Further doubt concerning a PT-interpretation of near-zero slopes comes from findings that reveal flatter or missing RT-slopes with figure stimuli in a standard mental rotation task (Amorim et al., 2006), as well as in a hand laterality identification task when a palm view of the human hand is presented (Ionta and Blanke, 2009). Without going into the particular nature of the underlying mechanisms (e.g., embodiment), such findings suggest that the absence of RT-slopes should not be regarded as positive evidence to dismiss OT-accounts.

Perspective transformations vs. OT/AT-instructions can also be manipulated by using stimuli rotated in the depth plane (i.e., the plane for which PTs in OBT- and AIS-tasks have been postulated). In memory-based AIS-tasks this has consistently yielded different RT-profiles (Wraga et al., 2000). Using a visual AIS-task with laterality decisions, Keehner et al. (2006) obtained comparable results, finding, in addition, differential brain activation for PT- vs. OT-instructions, supporting the assumption of processing differences between both. Although the experimental setup in Keehner et al. (2006) confounds rotations in the depth plane with incompatibility, this confound was, on average, equal for the PT- and OT-instructions. This seems a promising approach to gain further insight into the processes invoked by different transformation instructions (for other examples see Zacks et al., 2003; Tadi et al., 2009; Wraga et al., 2010).

### EVIDENCE FOR SPONTANEOUS PERSPECTIVE TRANSFORMATIONS

Whereas the evidence reported so far does not seem compelling in ruling out alternative transformation accounts of OBT- and AIS-performances, more convincing evidence for PTs comes from research in which observers make laterality decisions regarding their current perspective on a visual scene, showing that task performance suffers interference from the depicted avatar’s perspective. More specifically, Zwickel (2009; also Zwickel and Müller, 2013) presented animations of simple geometrical shapes and asked participants to make left/right decisions – from their own perspective – about briefly presented dots. Performance in this task was impaired when the laterality of the dot mismatched its laterality regarding the perspective ascribed to the animated figure. Obviously, such a finding could not result from a confounding with spatial compatibility, because responding always corresponded to the laterality of the critical feature from the observer’s perspective. It also does not seem reasonable to assume that OTs operated on the avatar-stimulus itself. The fact that no similar interference effects for laterality decisions about OBT-stimuli from their own perspective (i.e., which-side-task) were found, suggests that ascriptions of agency and/or embodied processing of the stimuli may be a prerequisite for spontaneous perspective taking (for discussions e.g., Kessler and Thomson, 2010; Kockler et al., 2010; Surtees and Apperly, 2012). This line of research seems interesting to pursue, as it could build a bridge to research on perspective conflicts and interference effects in cognitive (May, 2004, 2007; Wang, 2004; Kelly et al., 2007; Keehner and Fischer, 2012), as well as emotional and social perspective taking (Vogeley et al., 2004; Decety and Jackson, 2006; Duran et al., 2011; Mazzarella et al., 2013).

## CONCLUSION

Our review reveals that there is less support for the assumption that visual perspective taking is based on observer-based PTs than one would believe when looking at the literature. The foregoing analysis of OBT- and AIS-studies using laterality judgments (and these are the majority of studies) reveals a quite complicated research situation with different problems standing in the way of a PT-account of visual perspective taking. On the one hand, OBT- or AIS-studies using laterality judgments have problems to separate spatial incompatibility costs from transformation costs, making compatibility a potential alternative explanation for some of the findings. On the other, there is at least some evidence that spatial transformations play a role in visual perspective taking, but little evidence that PT-accounts of this role are more convincing than OT-accounts in case of OBT-performances, or AT-accounts in case of AIS-performances.

## RECOMMENDATIONS

In order for future research to further close in on the mechanisms underlying visual perspective taking the following methodological recommendations might be helpful:

(1) When using OBT- or AIS-tasks in combination with laterality decisions, take effective measures to control for spatial compatibility.
(2) The measure taken should allow disentangling the independent contributions of spatial transformation and spatial conflict costs (for steps in this direction see Gardner and Potts, 2011, or May and Wendt, 2012, similar measures are conceivable for AIS-tasks).
(3) To exclude compatibility influences altogether, non-lateralized spatial judgments should be preferred; for example, same/different decisions in OBT-tasks, force-choice decisions, object naming or object counting in AIS-tasks. When using such tasks, look out and control for possible hidden laterality influences (e.g., uneven spatial distribution of features/objects in both tasks).

## Conflict of Interest Statement

The authors declare that the research was conducted in the absence of any commercial or financial relationships that could be construed as a potential conflict of interest.
